# *Lactobacillus rhamnosus GG* (ATCC 53103) for the Management of Infantile Colic: A Randomized Controlled Trial

**DOI:** 10.3390/nu12061693

**Published:** 2020-06-05

**Authors:** Francesco Savino, Paola Montanari, Ilaria Galliano, Valentina Daprà, Massimiliano Bergallo

**Affiliations:** 1 Department of Pediatrics, S.S.D. Subintensive Neonatal Care, Children Hospital “Regina Margherita”, A.U.O. Città della Salute e della Scienza di Torino, 10126 Torino, Italy; 2 Dipartimento delle Scienze di Sanità Pubblica e Pediatriche, Università degli Studi di Torino, Scuola di Medicina, 10126 Torino, Italy; paola.montanari@unito.it (P.M.); ilaria.galliano@unito.it (I.G.); valentina.dapr@yahoo.it (V.D.); massimiliano.bergallo@unito.it (M.B.)

**Keywords:** *Lactobacillus rhamnosus* (ATCC 53103), infantile colic, *Lactobacillus*, total bacteria, faecal calprotectin

## Abstract

Background: The aim of this study was to investigate the efficacy of *Lactobacillus rhamnosus* ATCC 53103 together with the maternal diet avoidance of cow’s milk in treating infantile colic. Methods: Forty-five colicky breastfed infants were consecutively randomized to receive *L. rhamnosus* for 28 days at a dosage of 5 × 10^9^ cfu per day or placebo. Faecal samples were collected from each subject before starting supplementation and at the end of the study period and were immediately analysed. Faecal calprotectin was detected via a quantitative assay. The total bacterial load and selected bacterial species were evaluated using real-time TaqMan PCR. Results: After supplementation for 28 days with *Lactobacillus rhamnosus* ATCC 53103, median full-force daily crying was reduced (104 versus 242 min, *p* < 0.001) and the values of faecal calprotectin decreased significantly (*p =* 0.026). Furthermore, the probiotic increased the abundance of *Lactobacillus* (*p* = 0.048) and total bacteria (*p* = 0.040); all these effects were not observed in the placebo group. Conclusion: Infants treated with *Lactobacillus rhamnosus ATCC 53103* for 28 days, in association with the elimination of cow’s milk from the maternal diet, presented some interesting features related to the effect of this probiotic treatment: reductions in crying time and faecal calprotectin, with increased total bacteria and *Lactobacillus.* To validate these results, a double-blind, placebo-controlled trial on a larger cohort is required.

## 1. Introduction

In recent years, it has become clear that the composition of the gastrointestinal microbiota plays a crucial role in the health of the adult human host. The microbes ensure mutualistic symbiosis, providing the host with beneficial functions, such as the metabolism of nondigestible compounds and the supply of vitamins, while they also prevent colonization by pathogens and assist in the regulation of immunity [1]. The imbalance or disruption of the host microbiota, termed “dysbiosis”, has been linked to several functional gut disorders, such as inflammatory bowel disease, and, recently, also to infantile colic [2]. 

The exact causes of infantile colic are not fully understood, but some likely hypotheses are gut dismotility and food intolerance [3]. Growing evidence suggests that the gut microbiota could be also involved in colic: a higher amount of Coliforms and a lower amount of Lactobacilli have been found in colicky infants compared to in non-colicky subjects [4,5,6], and probiotic supplementation with *Lactobacilli reuteri* has been shown to be effective in treating the condition in some clinical trials of breastfed infants [7,8,9,10].

However, dysbiosis and imbalance in the taxonomic composition of the gut bacteria occurring during the early stages of gut development can induce modifications in the immunological and metabolic phenotype favoring the development of infantile colic [6], which is associated with low-grade systemic inflammation [11].

Specific bacterial species such as *Lactobacillus rhamnosus* (ATCC 53103) may have anti-inflammatory properties that may influence the gut microbiota and improve colic-related inflammation [12,13]. A greater understanding of the therapeutic benefit of *L. rhamnosus* on colic is warranted.

The possible link between infantile colic and atopy derives from two lines of evidence: the presence of gut microbiome dysbiosis reported in both conditions [2,4,5,6,14]; and the good clinical response to dietary interventions in infantile colic [15,16].

Thus, in the present study, we performed a randomized controlled clinical trial aimed to verify whether *Lactobacillus rhamnosus ATCC 53103* is effective in treating infantile colic of breastfed term infants and reducing gut inflammation by measuring faecal calprotectin levels and gut microbiota.

## 2. Materials and Methods 

### 2.1. Study Population

We carried out a randomized study from March 2018 to October 2019. This randomized trial involved 47 colicky breastfed infants, aged 10–60 days. All infants were examined by a pediatrician at the outpatient department of pediatrics of the University of Turin-Regina Margherita Children Hospital—Città della Salute e della Scienza di Torino.

The inclusion criteria were as follows: infants aged between 2 and 10 weeks, gestational age between 37 and 40 weeks, birth weight between 2700 and 4200 g, and exclusively breastfed. Subjects were excluded if they had clinical evidence of chronic illness or gastrointestinal disorders or if they had received antibiotics or probiotics in the week preceding recruitment.

Colic was diagnosed according to the Wessel definition [17] modified by Roma IV criteria [18], i.e., when infants had at least three episodes of unexplained full-force crying lasting more than three hours per day on at least three days a week for at least one week. 

All parents provided written informed consent to the inclusion of their infants in the study. The protocol was approved by the Ethics Committee of the Azienda Ospedaliera, OIRM S. Anna—Ospedale Mauriziano in Turin, Italy (19 November 2016. Delibera prot. 7/ 2017). ISRCTN16554977.

### 2.2. Study Design 

At enrollment, each infant underwent a medical examination by a pediatrician (F.S.), and parents completed a questionnaire to obtain data concerning type of delivery, birth weight, and gestational age. The pediatrician asked the parents to report full-force crying by means of a well-validated three-day diary before the faecal sample collection [19]. A diet eliminating cow’s milk and dairy products for the breastfeeding mother was requested. The mean daily minutes of crying were calculated as a sum of crying, fussing, and unsuitable crying using a parental diary.

The administration was randomized using 5 drops of *Lactobacillus rhamnosus* (ATCC 53103) (5 × 10^9^ colony for units per day) or placebo for a 28-day study period. Randomization was performed by the random-digit method, on the basis of a computer-generated numbers. We used a two treatments randomization scheme with a random block of varying size (Stata Statistical Software: Release 9. StataCorp LP, College Station, TX, USA. Ralloc Procedure). To evaluate tolerance, growth parameters (weight gain per day), symptoms of digestive intolerance (constipation, regurgitation, or vomiting), and frequency of adverse events during the treatment period were evaluated daily. Adverse events, defined as illnesses, signs, or symptoms that occurred or got worse during the course of the study, were assessed through parental reporting data daily records. A pediatrician allocated infants the following available product on entry into the trial, and each patient received the study product directly from our Hospital. Probiotic study product consisted of a suspension of freeze-dried *L. rhamnousus ATCC53103* in a mixture of mais oil and mono and diglyceride oil supplied in a 5-mL dark bottle fitted with a dropper cap. The placebo was an identical mixture of mais oil and mono and diglyceride oil in appearance and taste but without probiotic. Both formulations were administered during the morning in 5 drops, once a day, 30 min before the feed, for a period of 28 days.

### 2.3. Sample Collection

About 5–10 g of faeces was collected from each subject directly from the diaper and immediately analysed for calprotectin values. Each sample was stored at −80 °C immediately after collection, in a numbered, screw-capped plastic container, until they were processed via real-time PCR. 

The person carrying out laboratory tests was blind to the identities of the infants.

#### 2.3.1. DNA Extraction

Before extracted DNA, stools were diluted with ultrapure H_2_O 1/10 and vortexed. Then, DNA was extracted as follows: 400 μL of supernatant was added to 400 μL of lysis buffer (400 mM Tris-HCl, pH 7.5, 500 mM NaCl, 50 mM EDTA, 1% SDS), incubated at room temperature for 90 min, and then centrifuged at 14,900× *g* for 5 min. The supernatant was mixed with an equal amount of phenol–chloroform and centrifuged at 14,900× *g* for 10 min. The supernatant was then mixed with an equal amount of isopropanol and incubated at −80 °C for 30 min for precipitation. After centrifugation at 14,900× *g* for 5 min, the pellet was washed with 70% ethanol, centrifuged at 14,900× *g* for 5 min, dried, and resuspended in 20 μL of ultrapure H2O. DNA extractions were quantified using a NanoDrop Lite spectrophotometer Thermo Scientific (Paisley PA4 9RF, Inchinnan, UK) and were diluted to the desired concentrations prior to each experiment.

#### 2.3.2. Faecal Calprotectin Analysis 

For the determination of faecal calprotectin, we used our department’s rapid test, the BÜHLMANN Quantum Blue^®^ Calprotectin High Range (Schönenbuch, Switzerland). This test allows for the quantitative determination of faecal calprotectin through a sandwich immunoassay with a measurement range between 100 and 1800 µg/g.

#### 2.3.3. Real-Time PCR 

Specific TaqMan real-time PCR for *Escherichia coli* (BioMole srl Italy, To. cod., PP-BioMole-080), *Bifidobacterium* spp. (BioMole. cod., PP-BioMole-082), and *Lactobacillus* spp. (BioMole. cod., PP-BioMole-081) was used as recommended by the manufacturer.

The broad-range real-time PCR PP-BioMole-083 assay was used as recommended by the manufacturer. Thermal cycling was performed using the ABI PRISM 7500 detection system (Applied Biosystems) as follows: 2 min at 50 °C for the uracil-*N*-glycosylase reaction, 2 min at 95 °C for denaturation, followed by 10 cycles of 15 s at 95 °C, 30 s at 55 °C, and 30 s at 72 °C, followed by 35 cycles of 15 s at 95 °C and 1 min at 60 °C [20].

### 2.4. Statistical Analysis

The continuous variables are reported with mean ± SD, median and interquartile range (IQR); the categorical variables are reported with frequencies and percentages. 

To examines if continuous variables are normally distributed, we use the Kolmogorov–Smirnov normality test (K–S). 

Sample size was calculated on the basis of the finding of previous studies [6,7] using a difference between groups of a 50-min reduction in daily average crying time, which was considered a clinically relevant difference. With α = 0.05, β 0.20 and an estimated standard deviation (SD) within groups of 55 min, 20 patients were needed in each group 

We used a paired *t*-test for paired sample to compare means from the same group at different times in parametrical variables, and we used the Wilcoxon signed-rank test to compare median ranks from the same group at different times in non-parametrical variables. 

We used the Student’s *t* test for an independent sample to evaluate differences between the means of the two groups in parametrical variables, and we used a Mann–Whitney U test to evaluate differences between the medians of the two groups in non-parametrical variables. 

All tests were 2-tailed and considered significant at values of *p* < 0.05.

The data were subjected to statistical analysis using the statistical software SPSS 24.0 (IBM SPSS Statistics for Windows, Version 24.0. Armonk, NY, USA: IBM Corp.), while sample size calculation was performed by NCSS-PASS 2000 (Number Cruncher Statistical Systems, Kaysville, UT, USA).

## 3. Results

A total of 47 infants were enrolled: 26 infants in the *L. rhamnosus* group and 21 infants in the placebo group out of 86 infants who met the inclusion criteria. The data of the type of delivery, gender, age at enrolment, and birth weight did not differ between the two groups. The baseline characteristics of the two study groups of infants involved in the study are shown in Table 1. In the *L. rhamnosus* group the four-week follow-up was completed by 24 subjects, two subjects did not complete the study period for reasons not related to treatment, while all placebo group subjects completed the study. There were no study-product-related adverse effects during the study period in both groups of infants. No differences were found between groups neither in median gain weight per day (placebo 29.9 IR = 19.4 versus *L. rhamnosus* 34.3 IR = 16.1; *p* = 0.140). There are no differences between groups in crying and fussing at Day 0 (Placebo 247.95 ± 19.6, *L. rhamnosus* 242 ± 39.4, *p* = 0.310).

### 3.1. Clinical Data: Crying and Fussing

The normal distribution of the variables is checked by the Kolmogorov–Smirnov test (K–S test). This test confirms that the *L. rhamnosus* variable at Day 0 and the *L. rhamnosus* variable at Day 28 are normally distributed (K–S Test *p* > 0.05) and the placebo variable at Day 0 and placebo variables at day 28 are normally distributed too (K–S test *p* > 0.05). We use the paired sample t-test to evaluate the differences between the means of *L. rhamnosus* variable at Day 0 (242.0) and the means of the *L. rhamnosus* variable at Day 28 (104.7) and the difference is statistically significant 137.3, *p* = 0.001. We use the paired sample t-test to evaluate the differences between the means of the placebo variable at Day 0 (247.9) and the means of the placebo variable at Day 28 (239.6) and the difference is not statistically significant 8.3, *p* = 0.106 (Table 2a).

We use the Independent Samples *t*-test to evaluate the differences between the means of Placebo variable at Day 28 (239.6) and the means of *L. rhamnosus* variable at Day 28 (104.7) and the difference is statistically significant 134.9, *p* = 0.001 (Table 2, Table 3).

### 3.2. Fecal Calprotectin

The variables are normally distributed (K–S test *p* > 0.01) so we use the paired sample t-test to evaluate the difference between the mean of the two groups. In the *L. rhamnosus* group, the difference between the mean of Calprotectin Day 0 and the mean of Calprotectin Day 28 is −119.53 and that is statistically significant (*p <* 0.05). In the placebo group, the difference between the mean of Calprotectin Day 0 and the mean of Calprotectin Day 28 is −10.41 and that is not statistically significant (*p* > 0.05) (Table 4).

### 3.3. Microbiological Analysis

**Figure 1:**
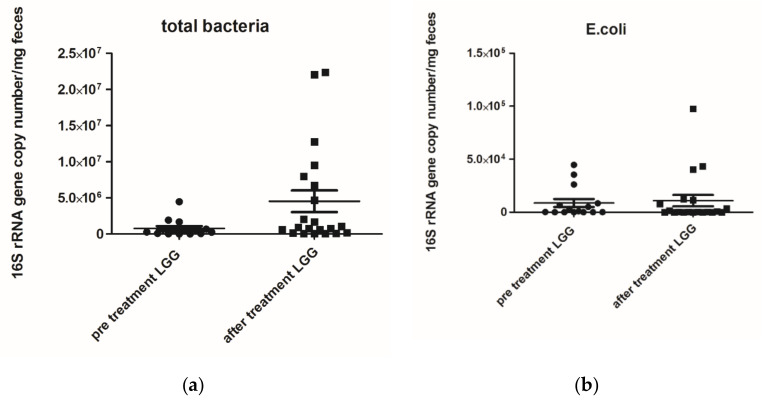
(**a**) Total bacterial genome (mg/faeces) pre- and post-treatment with *Lactobacillus rhamnosus* ATCC 53103 (LGG) *p* value 0.0400; (**b**) *Escherichia coli* (*E. coli*) 16S rRNA gene copy number (mg/faeces) pre- and post-treatment with *Lactobacillus rhamnosus ATCC 53103 (LGG) p* value 0.8533. Footnote: The data are represented as black dots. 16S rRNA gene copy numbers were assessed by real-time PCR and represented 16S rRNA gene copy number/mg feces. The statistical significance was calculated by the Mann–Whitney *t*-test.

**Figure 2:**
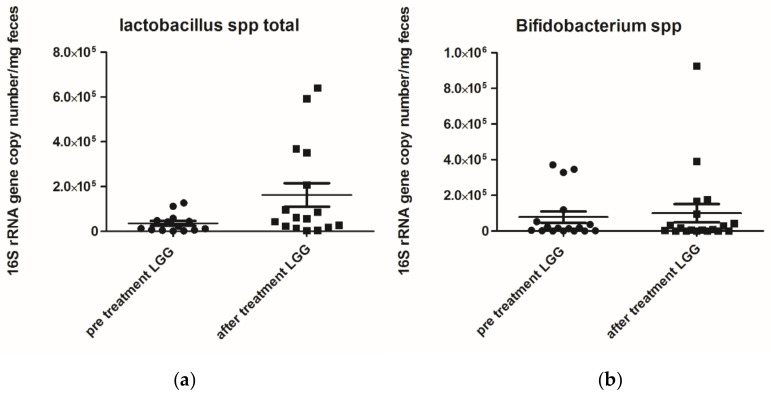
(**a**) *Lactobacilluss* spp. 16S rRNA gene copy number (mg/faeces) pre- and post-treatment with *Lactobacillus rhamnosus* ATCC 53103 (LGG) *p* value 0.0483; (**b**) *Bifidumbacterium* spp. (mg/faeces) pre- and post-treatment with *Lactobacillus rhamnosus* ATCC 53103 (LGG) *p* value 0.8245. Footnote: The data are represented as black dots. 16S rRNA gene copy numbers were assessed by real-time PCR and represented 16S rRNA gene copy number/mg feces. The statistical significance was calculated by the Mann–Whitney *t*-test.

The microbiological analysis of faecal samples pre- and post-treatment with *L. rhamnosus* allowed us to obtain the following results: total bacteria were significantly increased after treatment (*p* = 0.04), but *E. coli* levels did not differ after treatment, as reported in Figure 1 and Figure 2. While the comparison of microbiological data of the infants who received placebo did not show any differences between Day 0 and day 28, as reported in Table 5. 

The faecal *Lactobacillus* spp. levels after treatment were significantly increased (*p* = 0.0483), while *Bifidumbacterium* spp. levels did not differ after treatment with *L. rhamnosus.*

## 4. Discussion

Our results suggest that probiotic intervention for 28 days with *L. rhamnosus* mitigates symptoms of infant colic, reducing daily crying time and faecal calprotectin levels in breastfed term infants. 

Some researchers studying these issues have argued that infant full-force crying behaviours have shared origins in disorders of “infant self-regulation” [21]. Other authors argue that this problematic behaviour may have two types of distinct causes: immaturity of intestinal function and motility and alteration in gut microbiota [3]. Furthermore, the unexplained bouts of crying that occur in around 20% of young infants have been attributed also to a possible food allergy disturbance and related pain, leading to the clinical designation of “infant colic” as a first sign of atopy [22]. Recent evidence suggests that infantile colic should be seen as the result of impairments in the gut microbiota with subsequent gut inflammation [2]. 

For these reasons, intervention with a probiotic such as *L. rhamnosus*, which is the most studied probiotic in this atopy field, may exert an insightful influence on gut immune system maturation and tolerance acquisition in the early life [12,13,23,24] and therefore play a pivotal role in the improvement of colic-related sympthoms. In this regard, *L. rhamnosus* administration has also been reported to induce tolerance in infants with suspected cow’s milk allergy [24].

The possible relationship between food intolerance and infantile colic comes from the presence of dysmotility with visceral hypersensitivity and dysbiosis. It is interesting to note that, in an animal model, *L. rhamnosus* can attenuate chronic visceral pain induced in the first period of life in rat. Kannampalli P. et al. hypothesized that a supplementation with LGG over time could alter some key brain neurotransmitters and biogenic amines that could be involved in pain modulation [25].

Regarding the immunological origin of infantile colic and role of possible allergens, it has been reported that cows’ milk proteins in breast milk or infant formula, could be one cause of colic [26]. 

In fact, cow proteins from the mother’s diet are hypothesized to pass into the breast milk and provoke an allergic response and symptoms of colic in some infants [27], and dietetic treatment should be a possible therapeutic approach [27,28].

Lundelin K et al. reported that neonate receiving *L. rhamnosus GG* perinatally tended to have decreased allergy prevalence later in life [29].

Furthermore, in addition to gut microbiota alterations [2], colic in infants is associated with low-grade systemic inflammation, as reported by Partthy et al. [11].

Fecal calprotectin is a direct measure of mucosal inflammation activity and becomes detectable when intestinal inflammation is still at an insufficient level to cause an increase in serum inflammation markers [30]. 

The calprotectin concentrations in faeces (faecal calprotectin) provide one marker for inflammatory bowel diseases and are sensitive and easy to measure [31]. Elevated calprotectin levels have been described in both adults and children with inflammatory bowel diseases, such as Crohn’s disease and ulcerative colitis, and can be used to assess the severity of inflammation in these patients [32]. It has been found that faecal calprotectin levels can indicate cow’s milk allergy and atopic disease, as well as gastrointestinal disorders [2,20,30,31]. The diagnostic value of calprotectin in infancy is also of growing interest [33,34].

In fact, we found a reduction in faecal calprotectin in infants treated with *Lactobacillus rhamnosus* as compared with the placebo group.

Until now, *Lactobacillus reuteri* DSM 17938 has been considered for the management of breastfed colic infants [35], while data on the benefit of other probiotic strains, such as *L. rhamnosus* were limited on infantile colic and were therefore analysed in the present trial. 

This study expands the current knowledge on probiotics, showing that *L. rhamnosus* is also effective in reducing crying time due to infantile colic. 

We recently observed that colicky infants treated with *Lactobacillus reuteri* for 30 days had an increased Forkhead box P3 FOXP3 concentration, resulting in decreased T helper cell 17/Regulatory T cell (Th17/Treg) ratio and faecal calprotectin levels with improved colic symptoms [20]. 

*Lactobacillus rhamnosus* GG components exert an anti-inflammation effect on epithelial cells such as mouse macrophage with a protective effect [36]. 

For these reasons, we have tested this specific bacterial species such as *L. rhamnosus*, unlike conventional probiotics, that may have anti-inflammatory properties that may influence the gut microbiota, reducing inflammation and alleviating colic.

Interestingly, it had been reported that the early use of prebiotics and probiotics with *L. rhamnosus* supplementation may alleviate symptoms associated with crying and fussing in preterm infants [37], but not in a population of term breastfed infants as in the present study.

Since the compositional growth of *Bifidobacterium* and *Lactobacillus* in the gut microbiota appears to be related with colicky symptoms [4,5,6], we also investigated the microbiological data of total bacteria, *Bifidobacterium*, *Lactobacillus*, and *E. coli* levels.

Our findings show that faecal samples after 28 days of treatment with *L. rhamnosus* had increased levels of total bacteria and of *Lacobacillus* spp. 

It is interesting to note that, in a recent report by Lougman et al., using 16S rRNA sequencing, the combination of machine learning findings with associative relationships revealed the potential prognostic utility of infant gut microbiota analysis in order to predict subsequent infant crying problems [5,38]. This could open new opportunities for a tailored approach to newborns with infantile colic and a family history of atopic disorders. 

### Study Limitations

Caution should be taken in using the data obtained from the present randomized study, and double-blind placebo studies are necessary to provide additional efficacy information.

The limitations of PCR protocols in the analysis of gut microflora: The conserved nature of ribosomal genome sequences across the different genera of bacteria and the high sensitivity of PCR allow for the detection of bacterial DNA even when present in extremely low concentrations and without the need for the isolation of organisms of interest. The exceptional sensitivity of PCR and the eubacterial nature of the 16S rRNA gene target also allow for the generation of false positive results when even a minute amount of nonviable bacterial DNA contaminates the enrichment media, the reagents, or the sample during collection and processing. There is increasing interest in and acceptance of the use of broad-range 16S rDNA PCR for routine diagnostic purposes in the clinical microbiology laboratory. There is no single conserved sequence for the use of broad-range PCR. There are different conserved regions within the 16S rRNA gene [39], and, therefore, there are different primer sequences that can be used for broad-range 16S rDNA PCR. Despite being broad-range primers, it is unlikely that any primer set will amplify all bacteria. Broad-range PCR should be standardised and controlled prior to use. Furthermore, it must be considered the potential overestimation of bacterial species due to variations in 16S rRNA gene copy numbers.

Molecular methods need specialized personnel and are cost-intensive. Diagnostic pathways that integrate molecular methods in routine care are lacking. Interpretation of studies that evaluate PCR-based methodology is hampered by the lack of a gold standard. On one hand, conventional culture may underestimate the presence of bacteria due to growth inhibition by antimicrobials. Molecular methods may also be compromised by contamination, either by carry over. Currently, molecular methods are unlikely to replace conventional blood cultures; they are resource-intensive, need specialized personnel, and have a higher hands-on time. Most importantly, they are currently not able to provide data on antibiotic susceptibility.

Despite its small sample size, our study suggests that *L. rhamnosus* treatment is associated with a reduction in faecal calprotectin levels in a discrete amount. However, in order to confirm our findings, further studies including a double-blind placebo control group should be performed. This could allow us to exclude age effects on the improvement. 

## 5. Conclusions

Infants treated with *Lactobacillus rhamnosus ATCC 53103* for 28 days, in association with the elimination of cow’s milk from the maternal diet, presented some interesting features related to the effect of this probiotic treatment: reductions in crying time and faecal calprotectin levels, with increased total bacteria and *Lactobacillus* spp.

Our findings also show that *L. rhamnosus* could represent a new tailored therapeutic approach for this common disturbance in early life. To confirm these results, a double-blind, placebo-controlled trial on a larger cohort is required.

## Figures and Tables

**Table 1:** Characteristics of the two study groups of infants involved in the study: *Lactobacillus rhamnosus* (ATCC 53103) and placebo.

	*L. rhamnosus* (*n* = 24)	Placebo (*n* = 21)	*p* Value
Type of delivery:			
vaginal/caesarean	14/10	12/9	*p* > 0.05 ^#^
Age at enrolment (days ± s.d.)	37.9 ± 15	41.8 ± 17	*p* > 0.05 *
Gender			
Female, n (%)	10 (41.6)	9 (42.8)	*p* > 0.05 ^#^
Male, n (%)	14 (58.4)	12 (57.2)	*p >* 0.05 ^#^
Birth weight (g ± s.d.)	3520 ± 450	3290 ± 390	*p* > 0.05 *
Weigth at day 0 age (g ± s.d.)	4270 ± 420	4080 ± 405	*p* > 0.05 *
Weight gain per day (g)	34.3 IR = 16.1	29.9 IR = 19.4	*p* > 0.05 *
Crying and time at day 0 (m)	242 ± 39.4	247 ± 19.5	*p* > 0.05 *

^#^ Fisher’s test, * Mann–Whitney test.

**Table 2:** Crying and fussing time (mean minutes per day) in the *L. rhamnosus* and placebo group at Day 0 and Day 28.

Group	Day 0 Mean	Day 28 Mean	Difference between Means	*p* Value
*L. rhamnosus* (*n* = 24)	242.0	104.7	−137.3	0.001
Placebo (*n* = 21)	247.9	239.6	−8.3	*p* > 0.05

Paired sample *t*-test.

**Table 3:** Comparison of crying and fussing time (mean minutes per day) between the mean of *L. rhamnosus* Day 28 and the mean of placebo Day 28.

*L. rhamnosus* Day 28 Mean (*n* = 24)	Placebo Day 28 Mean (*n* = 21)	Difference	*p* Value
104.7	239.6	134.9	0.001

Independent sample *t*-test.

**Table 4:** Descriptive variable of fecal calprotectin (media, ±standard deviation), µg/g, in infant with colic at enrolment and after 28 days of subjects supplementated with *L. rhamonosus* or placebo.

Fecal Calprotectin	Day 0	Day 28	Mean Difference	*p* Value
*L. rhamnosus* (*n* = 24) *	255.4 (±131.58)	135.86 (±108.22)	−119.53	0.026
Placebo (*n* = 21) *	204.91 (±143.49)	194.50 (±176.98)	−10.41	*0.821*

* The variables are normally distributed (* K–S test = Kolmogorov–Smirnov test *p* value > 0.05).

**Table 5:** Certain bacterial species of gut microbiota (mean ± standard deviation) *genome/mg feces*, in a group of infants’ *Lactobacillus rhamnosus GG* (ATCC 53103) or placebo at enrolment Day 0 and after 28 days of supplementation.

	*L. rhamnosus* Group (*n* = 24)	*p* Value	Placebo Group (*n =* 21)	*p* Value
**Total Bacteria**				
Day 0	293,576 (±1,433,471)	0.040 ^#^	16,110 (±64,580)	*p* > 0.05
Day 30	409,845 (±721,248)		13,655 (±14,445)	
*E. Coli*				
Day 0	318 (±34)	*p* > 0.05	156 (±1169)	
Day 28	3123 (±1864)		131 (±4235)	*p* > 0.05
*Lactobacillus*				
Day 0	28,857 (1 ± 55,662)	0.048 ^#^	3835 (±20,023)	*p* > 0.05
Day 28	79,570 (±298,763)		4625 (±28,606)	
*Bifidobacteria*				
Day 0	20,350 (±432,389)	*p* > 0.05	10,884(3 ± 9032)	*p* > 0.05
Day 28	66,043 (±503,326)		10,127(±26,786)	

Mann–Whitney *t*-test; # *p* < 0.05.

## Abbreviations

L. rhamnosus	Lactobacillus rhamnosus ATCC 53103
SD	Standard deviation
IC	Infantile colic
IQR	Interquartile range
PCR	Polymerase Chain Reaction
*g*	grams
*m*	minutes
cfu	Colony-forming unit
TaqMan RT-PCR	TaqMan Real-Time reverse-transcription
